# Friedel–Crafts Crosslinked Highly Sulfonated Polyether Ether Ketone (SPEEK) Membranes for a Vanadium/Air Redox Flow Battery

**DOI:** 10.3390/membranes4010001

**Published:** 2013-12-30

**Authors:** Géraldine Merle, Filipoi Carmen Ioana, Dan Eugen Demco, Michel Saakes, Seyed Schwan Hosseiny

**Affiliations:** 1 Department of Mechanical & Industrial Engineering, Concordia University, Montreal, QC H4B 1R6, Canada; 2 Faculty of Dentistry, McGill University, 3640 University street, Montreal, QC H3A0C7, Canada; E-Mail: geraldine.merle@mail.mcgill.ca; 3 Functional and Interactive Polymers, DWI RWTH Aachen University, Forckenbeckstrae 50, D-52074 Aachen, Germany; E-Mails: horvat@dwi.rwth-aachen.de (F.C.I.); demco@mc.rwth-aachen.de (D.E.D.); 4 Department of Physics, Technical University of Cluj-Napoca Memorandumului 28, R-400114 Cluj-Napoca, Romania; 5 Magneto Special Anodes B.V., Calandstraat 109, 3125 BA Schiedam, The Netherlands; E-Mail: michel.saakes@wetsus.nl

**Keywords:** cation exchange membrane, redox flow battery, membrane technology

## Abstract

Highly conductive and low vanadium permeable crosslinked sulfonated poly(ether ether ketone) (cSPEEK) membranes were prepared by electrophilic aromatic substitution for a Vanadium/Air Redox Flow Battery (Vanadium/Air-RFB) application. Membranes were synthesized from ethanol solution and crosslinked under different temperatures with 1,4-benzenedimethanol and ZnCl_2_ via the Friedel–Crafts crosslinking route. The crosslinking mechanism under different temperatures indicated two crosslinking pathways: (a) crosslinking on the sulfonic acid groups; and (b) crosslinking on the backbone. It was observed that membranes crosslinked at a temperature of 150 °C lead to low proton conductive membranes, whereas an increase in crosslinking temperature and time would lead to high proton conductive membranes. High temperature crosslinking also resulted in an increase in anisotropy and water diffusion. Furthermore, the membranes were investigated for a Vanadium/Air Redox Flow Battery application. Membranes crosslinked at 200 °C for 30 min with a molar ratio between 2:1 (mol repeat unit:mol benzenedimethanol) showed a proton conductivity of 27.9 mS/cm and a 100 times lower VO^2+^ crossover compared to Nafion.

## 1. Introduction

Cation exchange membranes, such as Nafion and sulfonated polyarylethers, are frequently used in many electrochemical energy storage applications. These applications require highly conductive and stable membranes. The vanadium/air redox flow battery (Vanadium/Air-RFB) is one of the most promising energy storage technologies based on such membranes.

The Vanadium/Air-RFB is an electrochemical energy storage system engaging fuel cell and all vanadium redox flow battery technology [1,2,3,4]. It consists of two electrochemical half-cells, separated by an ion exchange membrane. One half-cell contains the vanadium redox couples, V^2+^/V^3+^, where the second half-cell contains either water vapor or air, depending on the charge or discharge modes of the system. The Vanadium/Air-RFB has been chosen as a special type of redox flow system, since it employs only vanadium in its 2+/3+ oxidation state, but not in the 4+/5+ oxidation state. Due to that, stability issues caused by the 4+/5+ oxidation state [5] can be neglected [1]. This is considered as one of the major advantages of the Vanadium/Air-RFB along with the weight and chemical reduction [1]. However, the crossover of water caused by vanadium crossover is one of the major issues in all vanadium redox flow batteries [6], leading to the dilution of the vanadium solution and, eventually, to a reduced performance. This would also play an important role in Vanadium/Air-RFB technology, since water in the air compartment will cause flooding of the electrode and a breakdown of the system [1]. Yet, many commercial membranes seem unsuitable for the Vanadium/Air-RFB, due to high crossover, as well as high production costs. Nafion, a perfluorosulfonated polymer, is one of the most stable and conductive membranes applied in electrochemical membrane reactors, such as fuel cells and all vanadium redox flow batteries. It shows excellent chemical stability and proton conductivity, but suffers from high vanadium crossover, since it has very broad and well interconnected hydrophilic channels through which vanadium ions can be transported [7]. Furthermore, the high costs of *ca*. $800/m^2^ would increase the overall costs of such a Vanadium/Air-RFB system. Due to the high price, many researchers have been searching for alternatives for Nafion.

Sulfonated non-fluorinated aromatic polymers received strong attention as a potential alternative to Nafion. To achieve high proton conductivities, one has to increase the sulfonation degree of such polymers up to a point where most of these sulfonated aromatic polymers become very much mechanically and chemically unstable. Low-cost polyarylates have shown exceptional chemical stability, especially polyether ketones, like polyether ether ketone (PEEK). Sulfonated PEEK (SPEEK) has shown good chemical stability in fuel cell tests [8], and it was also investigated in all vanadium redox flow batteries [7,9].

In all vanadium redox flow batteries, SPEEK was mainly used to re-cast on Nafion membranes to decrease the vanadium crossover, due to its glassy properties and the undefined and not well-interconnected proton conductive channels [9]. However, the micro-structure difference of SPEEK, which does not provide the distinct hydrophilic-hydrophobic separation, like in the Nafion, leads to lower proton conductivities. Yet, the proton conductivity can be improved by increasing the extended sulfonation time [10,11] or sulfonation temperature [11]. Hence, it might well be valuable to use an all SPEEK membrane for the Vanadium/Air-RFB application.

However, as was mentioned earlier, an increasing sulfonation degree will lead to a poor mechanical stability of the membrane, due to high swelling up to total dissolution in water. This would make the membrane not suitable for any separation technique using protic and polar solvents.

To overcome this issue, crosslinking of the polymer has been shown to be a suitable route to increase the mechanical stability of such highly sulfonated polymers [12]. Crosslinking is the association of polymer chains through an ionic or covalent bond and can be performed on three different pathways: (a) physical crosslinking (ionically) [13]; (b) irradiation-assisted crosslinking; and (c) chemically crosslinking. SPEEK crosslinking has been described for all three methods in the scientific literature [13,14,15,16,17,18,19,20].

However, all the mentioned crosslinking methods show major issues and challenges in terms of feasibility,environmental sustainability and efficiency. Yet, an interesting chemical crosslinking method has been reported by [21] using bishydroxymethyl aryls. In this study, the focus has been set on thermal initiated crosslinking of the SPEEK by bishydroxymethyl aryl compounds. The crosslinking only occurs at the ${\text{SO}}_{3}^{-}$ groups facing the same issue as in the case of many crosslinking methods. Based on bishydroxymethyl aryls as the crosslinker, also Linkous and Rhoden [22,23] reported the crosslinking of SPEEK via the Friedel–Craft alkylation route. This route was already discussed earlier by Yao *et al.* [24] on polystyrene. Linkous and Rhoden reported a crosslinking on the backbone of SPEEK with a higher proton conductivity. Crosslinking was reported to occur at 200 °C for ten minutes. In analogy to the work of Linkous and Rhoden, this paper present the crosslinking of SPEEK by 1,4-benzenedimethanol via Friedel–Crafts alkylation. In particular, this paper focuses on the crosslinking mechanism, the influence of the time and temperature of crosslinking on proton conductivity, swelling and vanadium permeation. In contrast to other crosslinking methods, the proposed Friedel–Crafts crosslinking represents a time-saving, environmental friendly procedure with only a little consumption of ${\text{SO}}_{3}^{-}$ groups. Furthermore, the membranes show high potentials for Vanadium/Air-RFB application, since the vanadium cross over through the membrane could be reduced by a factor of 100 compared with the Nafion membrane.

## 2. Experimental Section

### 2.1. Membrane Preparation

The preparation of highly sulfonated SPEEK was prepared following the procedure described elsewhere [11]. A 10%–15% weight solution of SPEEK in ethanol was prepared and filtered through a 20 μm filter to remove insoluble particles. For the crosslinking, 1,4-benzenedimethanol was added to the solution with a molar ratio between 2:1, 3:1 and 4:1 (mol repeat unit:mol benzenedimethanol). ZnCl_2_ is added as a catalyst (1% w/w) with respect to the dry weight of the SPEEK. Membranes were cast from this solution on a glass plate with a 0.47 mm casting knife and dried overnight under nitrogen. After drying, the membranes were heat treated in an oven with a temperature of 150 °C or 200 °C with a duration between 10 min and 2 h. After curing, the membranes were taken off the glass plate and washed in boiling Milli-Q water to rinse the ZnCl_2_ out of the membrane.

### 2.2. Proton Conductivity

Through plane proton conductivity, measurements were carried in an in-house built two-electrode cell. The cell had a Teon interior with two circular platinum electrodes, with a surface area of 0.785 cm^2^. Both electrodes were connected with two wires, one for carrying the current and one to act as the potential probe. The cell was connected to a frequency response analyzer (FRA; Autolab). The membranes were soaked prior to measurement (24 h) with 3 mol H_2_SO_4_. For the conductivity measurements, the soaked membranes were clamped between the two platinum discs, and AC impedance spectroscopy at a oscillation amplitude of 100 mV in the frequency range of 100 Hz to 10^5^ Hz was performed. The resistance of the membranes was calculated by fitting the impedance response to the equivalent circuit model [25]. The conductivity was calculated by Equation (1), where *σ* is the proton conductivity of the membrane in mS/cm, *l* is the thickness of the membrane in cm and R is the resistance of the membrane in Ω.

(1) $$\sigma =\frac{1}{l\times R}$$

### 2.3. Ion Exchange Capacity (IEC)

The ion exchange capacity (IEC) was determined through titration. The membranes in H+ form were immersed in 1 M NaCl solution for 24 h to transform the membrane into the sodium form. The NaCl solution was then back titrated with 0.1 M NaOH. The IEC was calculated by Equation (2), where IEC is the ion exchange capacity in meq/g (or mmol/g).

(2) $$\text{IEC}=\frac{\text{volume added NaOH}(mL)}{\text{dry weight membrane}(g)}\times \text{NaOH concentration}(\frac{\text{mmol}}{mL})$$

### 2.4. Water Uptake

To determine the water uptake, the dry weight, *m_dry_*, and the wet weight, *m_wet_*, of the membrane are measured. The swelling was calculated according to Equation (3).

(3) $$\text{Swelling}(\%)=100\times \frac{m_{\text{wet}}-m_{\text{dry}}}{m_{\text{dry}}}$$

### 2.5. Permeability of Vanadium Ions

**Figure 1:**
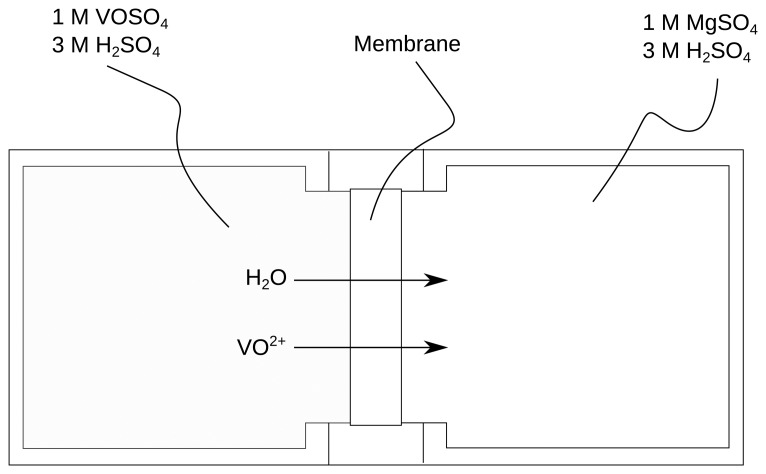
Schematics of a diffusion cell for vanadium permeation evaluation, indicating the VO^2+^ transport and the water transport due to the hydration shell of the VO^2+^.

The vanadium permeation was measured using a diffusion cell described elsewhere [26] and depicted in Figure 1. The diffusion cell consisted of two half cells with 75 mL volume each. The solutions were stirred continuously at a stirring speed of 300 rpm. Samples from the MgSO_4_ half cell of the diffusion cell were taken for UV-Vis Spectroscopy analysis to investigate the vanadium permeability. The permeability was calculated by Equation (4) [27].

(4) $$-\ln\;(1-\frac{C_{t}}{C_{0}})=\frac{P\times a}{l\times \upsilon }\times t$$

where *C_t_* is the vanadium concentration in the MgSO_4_ reservoir in mol/L, *C*_0_ the initial concentration of the vanadium in the vanadium reservoir in mol/L, *P* the permeability in cm^2^/s, *a* the area of the membrane exposed to the solution in cm^2^, *l* the thickness of the membrane in cm and *υ* the volume of the volume of the solution in both sides in mL. VO^2+^ was chosen, because it shows the highest permeability through Nafion after V^2+^ [28]. However, since V^2+^ is air-sensitive and difficult to maintain in its oxidation state, the 4+ oxidation state has been used to calculate the permeability.

### 2.6. Fourier Transform Infra-Red Spectroscopy

Fourier Transform Infrared Spectroscopy (FT-IR) measurements were done with the Frontier FT-IR from Perkin Elmer in order to determine the position of the crosslinking.

### 2.7. Pulsed Field Gradient Nuclear Magnetic Resonance Spectroscopy (PFG NMR)

Water self-diffusion measurement was performed at 70 °C on a Bruker minispec mq 20 MHz NMR spectrometer equipped with a pulsed gradient unit of maximum strength *G_x,max_* = 3.54 T/m. The direction of the gradient (*x*-axis) is set to be perpendicular to the direction of the static magnetic field (*z*-axis). The gradient strength was calibrated by measuring the self-diffusion coefficient of water (*D* = 3.235 × 10^−9^ m^2^ s^−1^) doped with CuSO_4_ at 40 °C. A pulsed-gradient stimulated echo (PGSE) sequence [30] was used with 14 gradient steps, from 0% to 90% of *G_x,max_* with a gradient-pulse length of *δ* = 0.5 ms. Echo time and the diffusion time, Δ, were 1 ms and 10 ms, respectively. For the gradient calibration, the Stejskal–Tanner relationship [30] was used, *i.e.*,

(5) $$ln(\frac{S(q,\Delta )}{S(0,\Delta )})=-q^{2}D(\Delta )\Delta_{\text{eff}}$$

where *D*(Δ*_eff_*) is the effective diffusion coefficient, *S*(*q*, Δ) is the signal intensity with applied gradient pulse, *S*(0, Δ) is the signal intensity in the absence of the gradient, *q*= *γδG_x_* is the wave vector and *γ* is the magnetogyric ratio. The effective diffusion time is defined as $\Delta =\Delta_{\text{eff}}-\frac{\delta }{3}$ [30]. For the measurement of the effective diffusion coefficient in the direction parallel and perpendicular to the membrane, a special sample holder from Teflon was constructed that allowed one to fix a stack of perpendicularly aligned membranes to the direction of the static magnetic field. The holder was then inserted into a NMR tube of 10 mm diameter. At 2 cm above the sample, a glass tube filled with water was mounted to keep 100% relative humidity inside the NMR tube.

## 3. Results and Discussion

### 3.1. Crosslinking SPEEK

**Figure 2:**
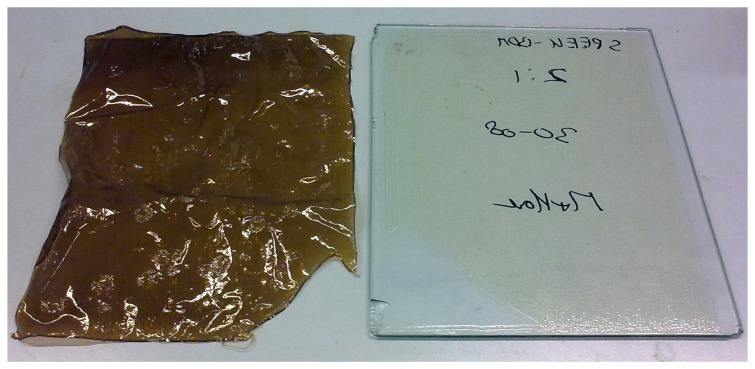
Crosslinked sulfonated poly(ether ether ketone) (SPEEK) membrane (**left**) and non-crosslinked SPEEK membrane cast on a glass plate (**right**).

Visual observation of the membrane before and after crosslinking at 150 and 200 °C were done (Figure 2). From the picture, it can be noticed, that cSPEEK appears in a different color than the non-crosslinked membrane. Crosslinked membranes exhibit a brown color, while the uncrosslinked membrane appears transparent. Moreover, it was observed that depending on the crosslinking conditions, the color varies from brown to black. This irreversible thermochromism may be an indication of a conformation change of the backbone, resulting from the crosslinking [31].

**Figure 3:**
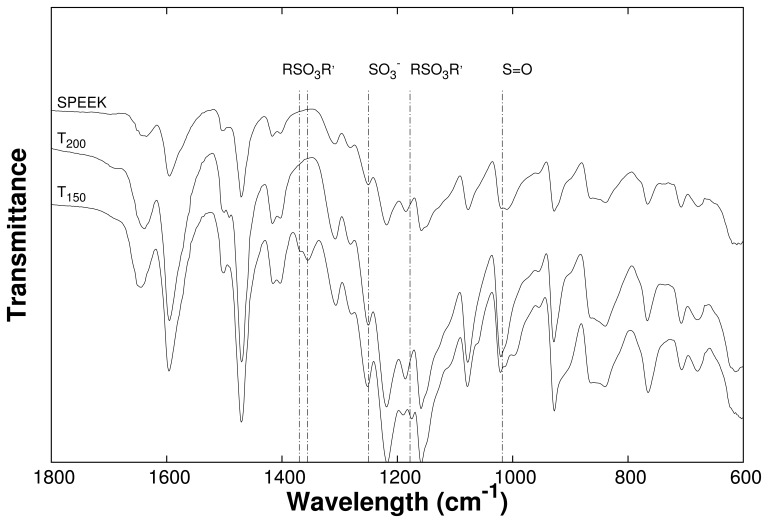
FT-IR of non-crosslinked and crosslinked SPEEK with the molar ratio SPEEK:crosslinker 3:1 (*T*_150_ = cSPEEK prepared at 150 °C, *T*_200_ = cSPEEK prepared at 200 °C). All crosslinked membranes were crosslinked for 60 min under the given temperatures.

The FT-IR spectra of crosslinked and non-crosslinked SPEEK are depicted in Figure 3. The crosslinking reaction was done under two different temperatures, 150 °C and 200 °C, as described in the Experimental Section. In Figure 3, no new peak appeared in the spectra of the membrane, which was crosslinked at 200 °C compared to the non-crosslinked SPEEK. However, in the case of the membrane crosslinked at 150 °C, three new peaks appeared.

Figure 3 shows that sulfonate ester (RSO_3_R') bands at 1370, 1356, 1178 wavelenght (cm^−1^) which appear in the cSPEEK membranes cured at 150 °C, but not at 200 °C. RSO_3_R' linkages are the result of a crosslinking reaction between the 1,4-benzenedimethanol and the sulfonic acid groups ( ${\text{SO}}_{3}^{-}$ at 1025 cm^−1^) of SPEEK, which had been reported to occur at even lower temperatures (135 °C) without catalyst, but a comparable crosslinker [21]. The disappearance of the sulfonate ester bonds at elevated temperatures might be due to the thermal instability of the sulfonate ester linkages, probably also affected by the catalyst, which had been described elsewhere [32,33].

**Figure 4:**
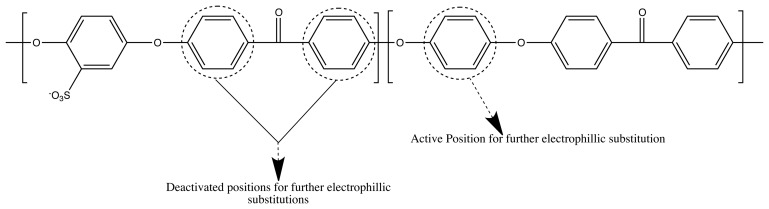
Activated and deactivated positions for an electrophilic substitution reaction on a SPEEK backbone.

The crosslinking position was reported to be in the sulfonated PEEK unit [22,23]. However, the possibility of a second electrophilic substitution, as the crosslinking reaction by Friedel–Crafts reaction, is drastically decreased by the electron withdrawing properties of the carbonyl group and the sulfonic acid group. This is depicted in Figure 4.

**Figure 5:**
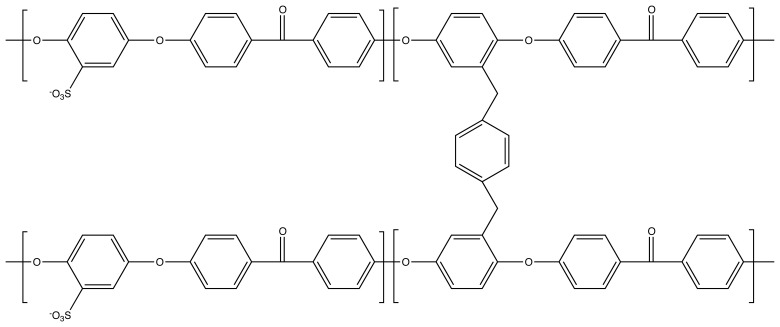
Activated and deactivated positions for an electrophilic substitution reaction on a SPEEK backbone.

Due to this reason, we also propose here a backbone crosslinking, but in a non-sulfonated SPEEK unit (Figure 5).

**Figure A1:**
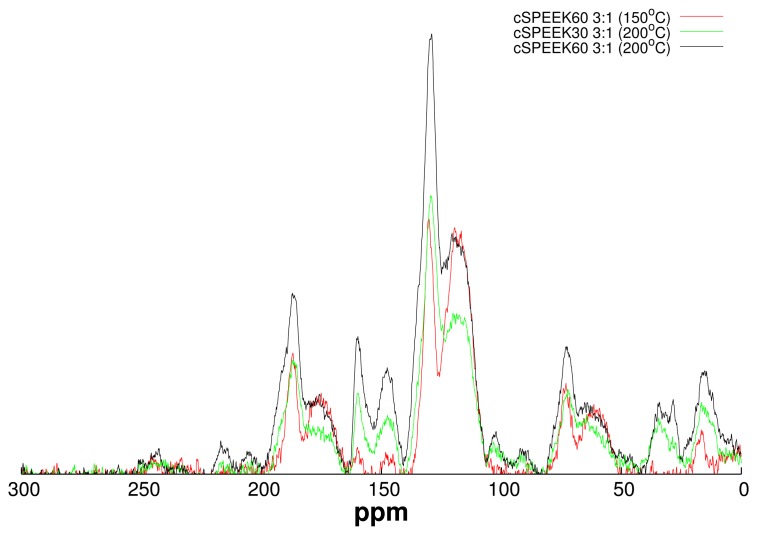
Solid State C^13^-NMR of crosslinked SPEEK membranes. Sample code: cSPEEK60 3:1 = crosslinked SPEEK with a crosslinking time of 60 min and a SPEEK:crosslinking ratio of 3:1).

Yet, we were not able to establish a solid proof for this assumption via solid state H-NMR, due to low resolution (see the Appendix, Figure A1), nor was this discussed by Linkous and Rhoden [22,23].

### 3.2. Channel Orientation by Water Diffusion Anisotropy

**Figure 6:**
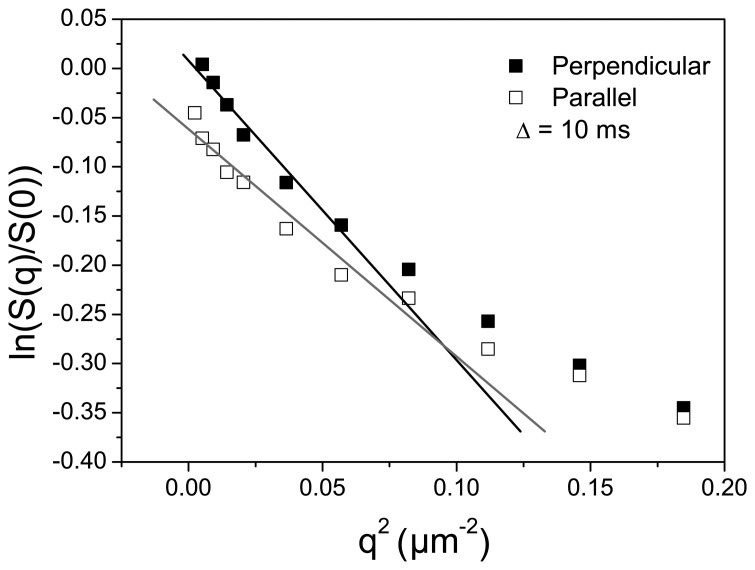
The dependence of the natural logarithm of normalized stimulated-echo amplitude as a function of *q*^2^ for cSPEEK membrane crosslinked at 200 °C for 60 min, for two orientations of the membrane plane relative to the direction of the magnetic field gradient.

The authors of a recent paper [34] measured quantitative bulk channel alignment in different Nafion membranes using ^2^H NMR directly on residually aligned absorbed D_2_O, and they found that channels are biaxially oriented in the membrane plane for extruded membranes, whereas they are uniaxially oriented perpendicular to the plane for dispersion-cast membranes. Furthermore, recently, a diffusion-exchange model with the assumption of two water pools was applied to describe the water transport in perfluorinated sulfonic acid (PFSA)/SiO_2_ nanocomposites [35]. The water diffusivity in-plane and through-plane in solution cast films was measured by NMR, revealing higher in-plane mobility. The anisotropy of the PFSA channels orientation reflected in the diffusivity anisotropy decreased with the increase in the nanofiller content. The dependence of the natural logarithm of normalized stimulated echo amplitude, ln[*S*(*q*, Δ)/*S*(0, Δ)] on the square of the wave vector (*q*^2^) is shown in Figure 6 for the SPEEK membrane crosslinked at 200 °C for 60 min, for the gradient perpendicular and parallel to the membrane plane and a diffusion time of Δ = 10 ms. The lines represent the best fit of the data, at low *q*^2^ range, where a “fast” diffusion occurs and from which apparent diffusion coefficients, namely D*_fast_*, can be extracted.

**Figure 7:**
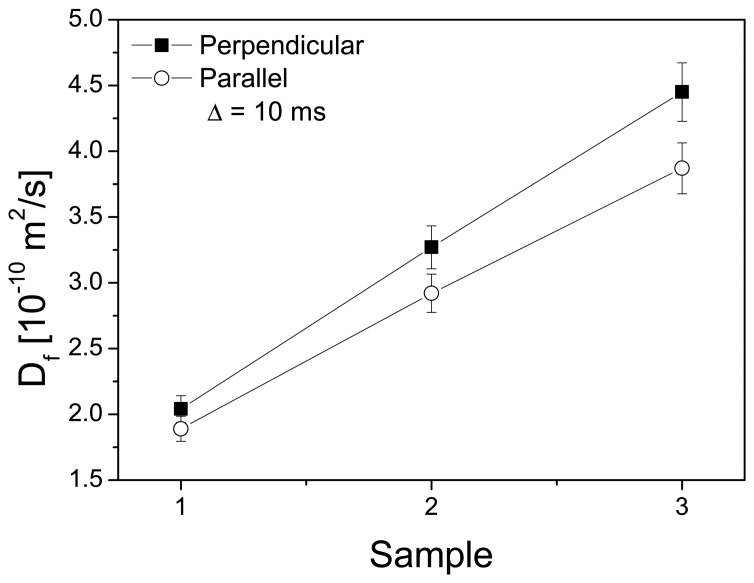
Water fast self-diffusion coefficients (*D_fast_*) of fully hydrated cSPEEK membranes with the magnetic field gradient parallel and perpendicular to the membrane plane. Sample code: Sample 1 = cSPEEK crosslinked at 150 °C for 60 min; Sample 2 = cSPEEK crosslinked at 200 °C for 30 min; and Sample 3 = cSPEEK crosslinked at 200 °C for 60 min.

Figure 7 depicts the water self-diffusion coefficients of cSPEEK membranes crosslinked at 150 °C for 60 min and 200 °C for 30 and 60 min and evidences the influence of the crosslinking treatment on the water diffusion process.

**Figure 8:**
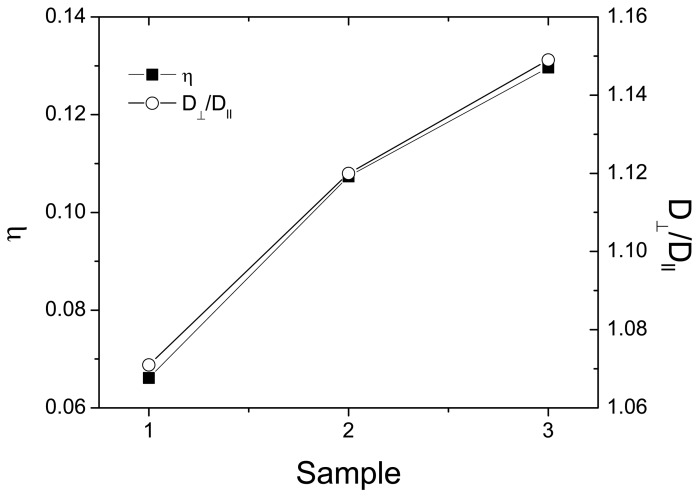
Anisotropy of water self-diffusion (*η*) and *D*_⊥_/*D*_∥_, for fully hydrated cSPEEK membranes. Sample code: Sample 1 = cSPEEK crosslinked at 150 °C for 60 min; Sample 2 = cSPEEK crosslinked at 200 °C for 30 min; and Sample 3 = cSPEEK crosslinked at 200 °C for 60 min.

The “fast” diffusion is characterized by a higher diffusion coefficient for the perpendicular orientation (through-plane) of THE gradient relative to the membrane plane than for the parallel one (in-plane). The SPEEK membrane crosslinked at 200 °C for 60 min shows the highest in-plane and through-plane diffusion among the studied membranes. The anisotropy of water diffusion in the cSPEEK membranes is defined as *η* = (*D*_⊥_ − *D*_∥_)/*D*_⊥_, where *D*_⊥_ corresponds to the through-plane diffusion coefficient and D_∥_ the in-plane diffusion coefficient, respectively. The anisotropy of water diffusion, as shown in Figure 8, increases about 100% with the temperature and time of crosslinking. In fully hydrated cSPEEK membranes, the anisotropy coefficient indicates a preferential orientation of water channels, perpendicular to the membrane plane. The increase value of water diffusion with the increase of the crosslinking temperature and time could be explained by the hydrophilicity of the channels, due to the preservation of the sulfonic acid groups. We should mention that a bimodal water diffusion is present in our samples. The water diffusion coefficient, called *D_slow_*, is more affected by the experimental errors and, therefore, is not shown in this work.

The increase of *D_fast_* in the crosslinked SPEEK membranes at elevated temperatures (200 °C) would indicate an improvement of proton conductivity through the membrane, which is beneficial for applications, such a vanadium redox flow batteries or fuel cells.

### 3.3. Swelling and Proton Conductivity

Table 1 depicts the swelling and proton conductivity of different membranes in water and in 3 M sulfuric acid as a function of crosslinking time, temperature and the composition of the membrane. Uncrosslinked samples cannot be used, since they can dissolve in water; hence, they cannot be used as a reference.

**Table 1:** Swelling and proton conductivity of different cSPEEK membranes with different SPEEK:crosslinker ratios (2:1, 3:1 and 4:1), for which the error of the conductivity data was reproducible in the range of 0.01%. In our experiments, Nafion reached at room temperature a proton conductivity up to 80.1 mS/cm, where a swelling of up to 26 wt% was reported elsewhere [36]. All swelling experiments were done at room temperature.

	**150 °C**				**200 °C**			
	
	**Swelling (%)**		**Proton Conductivity (mS/cm)**		**Swelling (%)**		**Proton Conductivity (mS/cm)**	
			
	**5 min**	**60 min**	**5 min**	**60 min**	**5 min**	**60 min**	**5 min**	**60 min**
cSPEEK 2:1	24 ± 7	23 ± 3	30.8	26.8	27 ± 10	32 ± 2	10.9	30.2
cSPEEK 3:1	42 ± 14	15 ± 1	36	8.4	35 ± 5	50 ± 8	13.3	36.3
cSPEEK 4:1	56 ± 4	22 ± 3	61.8	9	45 ± 6	32 ± 1	25.9	38.1

From Table 1, it can be concluded that proton conductivity and swelling are strongly influenced by the concentration of the crosslinker, the treatment temperature and time. However, simple trends cannot be identified easily. The swelling values for membranes crosslinked at 200 °C for 60 min showed an increase in the average compared to the swelling behavior of the same membranes treated at 150 °C for the same time. This is possibly due to the fact that the ${\text{SO}}_{3}^{-}$ groups were not affected negatively by the crosslinking reaction after 60 min at 200 °C, but at 150 °C. Indeed, at 150 °C and 60 min, the crosslinking took place at the ${\text{SO}}_{3}^{-}$ groups, leading to a sulfonate ester, as previously indicated by the FT-IR spectroscopy. This decreased the hydrophilic properties of the membrane, leading to a decrease of the swelling of the membrane. This fact is also reflected in Table 1, where the conductivity is compared with the swelling. At 200 °C, not only the swelling increased, but also the proton conductivity. This is probably due to the thermal instability of the sulfonate ester, which can turn back to its original form, *i.e.*, ${\text{SO}}_{3}^{-}$. At this point, it has to be pointed out, that the swelling behavior of the SPEEK membranes crosslinked for 60 min at 200 °C does not agree with the conductivity data for this series. This little deviation is most probably due to experimental errors. However, in order to choose a suitable membrane for the Vanadium/Air-RFB, swelling and conductivity need to be adjusted carefully. A highly conductive membrane may show, as well, high swelling, and by this, the risk of high vanadium crossover is given. Due to that, cSPEEK60 2:1 and cSPEEK60 3:1 would possibly represent suitable candidates for the Vanadium/Air-RFB application.

**Figure 9:**
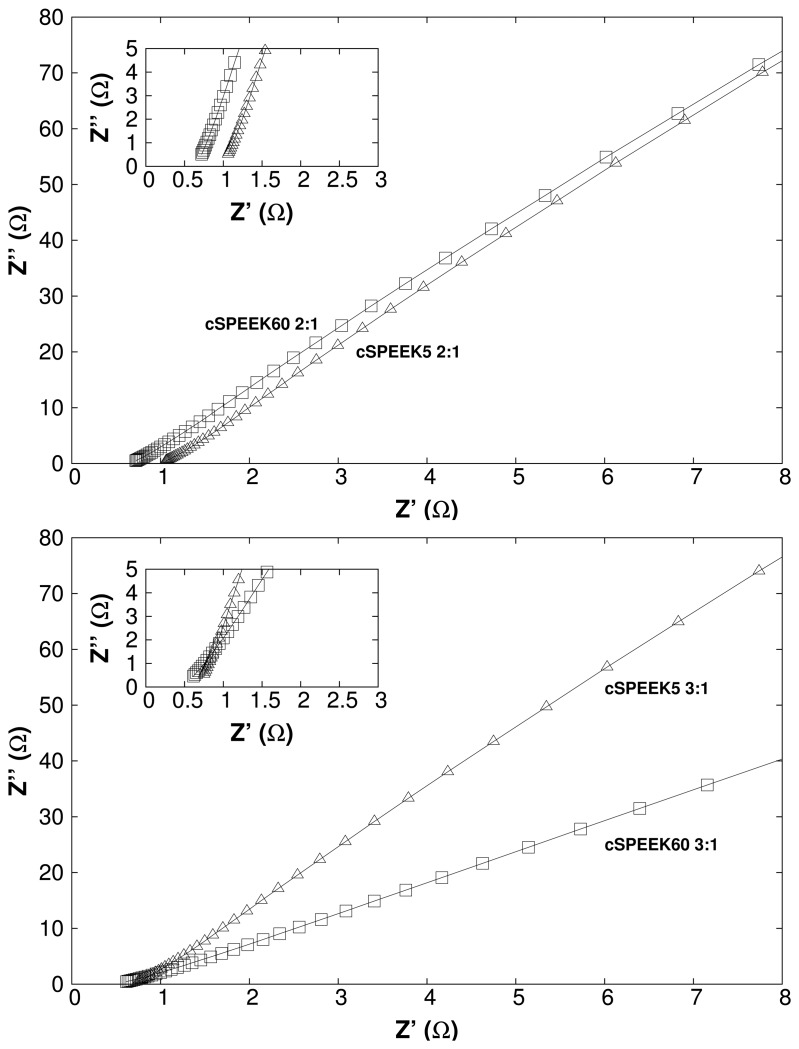
Electrochemical impedance spectra of crosslinked SPEEK series cured at *T* = 200 °C (sample code: cSPEEK60 2:1 = crosslinked SPEEK with a crosslinking time of 60 min and a SPEEK:crosslinking ratio of 2:1).

**Figure 10:**
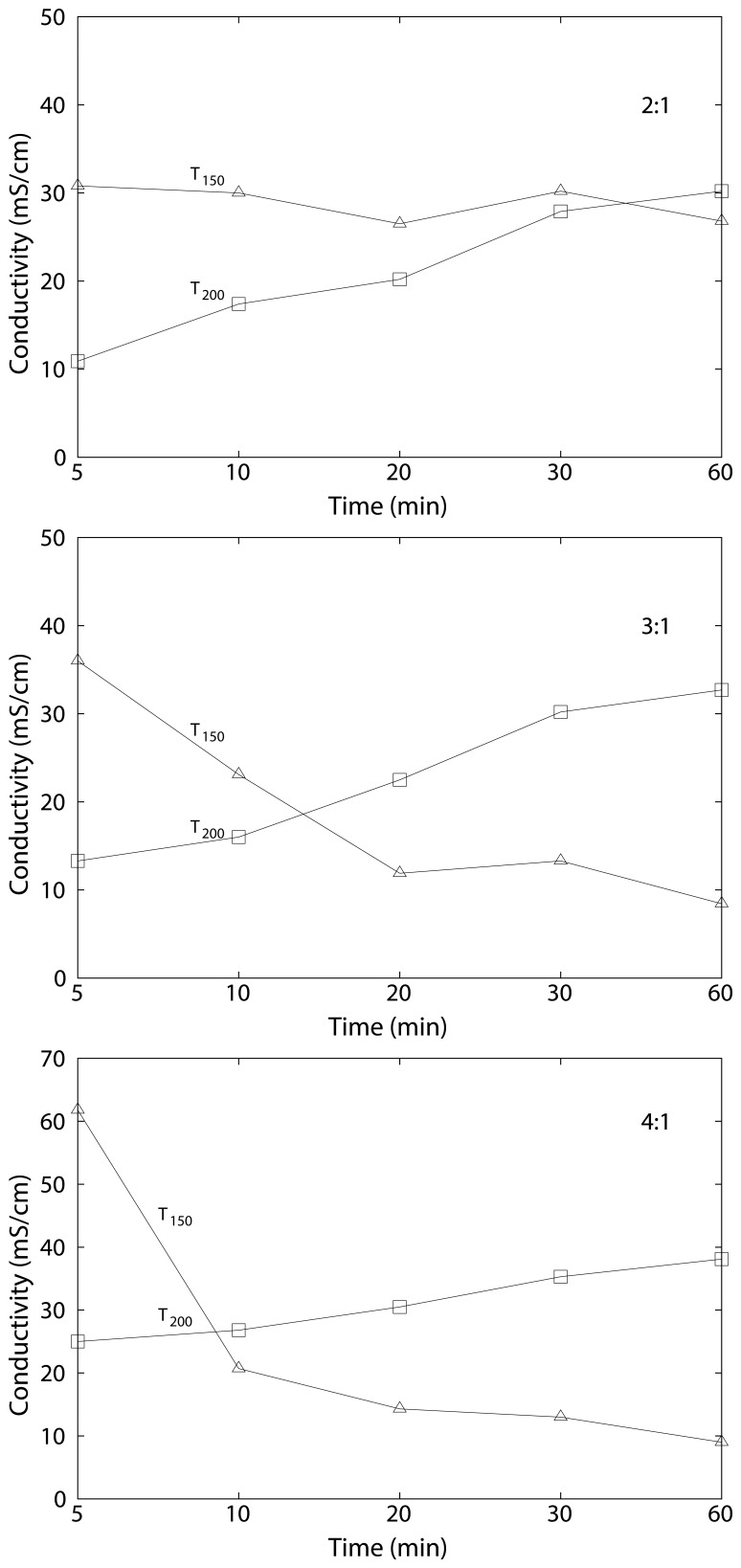
Conductivity of crosslinked SPEEK as a function of crosslinking time with different SPEEK:crosslinker ratios indicated in the upper right corner of the graphs (*T*_150_ = cSPEEK prepared at 150 °C, *T*_200_ = cSPEEK prepared at 200 °C).

**Figure 11:**
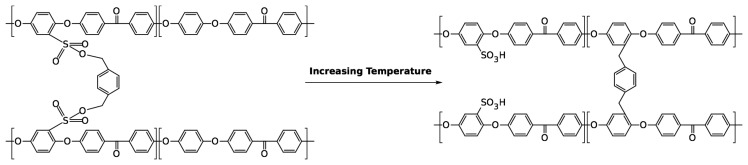
Crosslinking positions of the 1,4 Benzyl-di-methanol.

**Figure 12:**
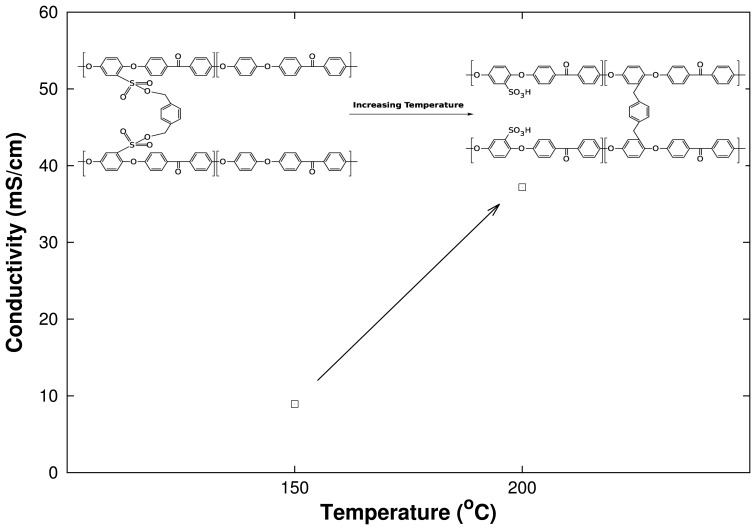
Dependency of crosslinking temperature and proton conductivity on one SPEEK membrane sample for a cSPEEK membrane with a SPEEK:crosslinker ratio of 3:1.

Figure 9 depicts the electrochemical impedance spectra of the at 200 °C-cured cSPEEK membranes for the 3:2 and 2:1 (SPEEK:crosslinker) series, where Figure 10 depicts the calculated conductivity behavior from the impedance data as a function of the crosslinking time and temperature. It can be seen that at 150 °C, the conductivity decreases with the time. For the crosslinking at 200 °C this behavior is inverted. This is related to the crosslinking position of the crosslinker, which can change spontaneously from the ${\text{SO}}_{3}^{-}$ group to the backbone of the PEEK repeat unit (Figure 11). To prove this theory, a SPEEK membrane was crosslinked in two steps. (1) SPEEK was crosslinked at 150 °C for 60 min; (2) The same membrane was further treated at 200 °C for another 60 min. The proton conductivity was measured via samples in between the two steps. The results regarding the proton conductivity of the membranes are depicted in Figure 12.

As illustrated in Figure 12, the ionic conductivity increases after the second step, *i.e.*, after exposition at 200 °C for 60 min. The resulting conductivities support the previous hypothesis of the sulfonate ester appearance at lower crosslinking temperature and the change of the crosslinking position of the 1,4-benzenedimethanol at higher temperatures and longer crosslinking duration.

The hypothesis of the heat conducted shifting of the crosslinking group also correlates with the ion exchange capacity (IEC) data. Indeed, IEC is directly related to the number of the ${\text{SO}}_{3}^{-}$ groups of the membrane. Its value mirrors the free amount of uncrosslinked ${\text{SO}}_{3}^{-}$ groups. It was found that the IEC of the membrane after 60 min at 200 °C was close to the initial value, *i.e.*, IEC for the non-crosslinked SPEEK (Table 2). Therefore, the IEC supports the hypothesis of the crosslinker shifting after a heat treatment.

### 3.4. Vanadium Permeability

**Figure 13:**
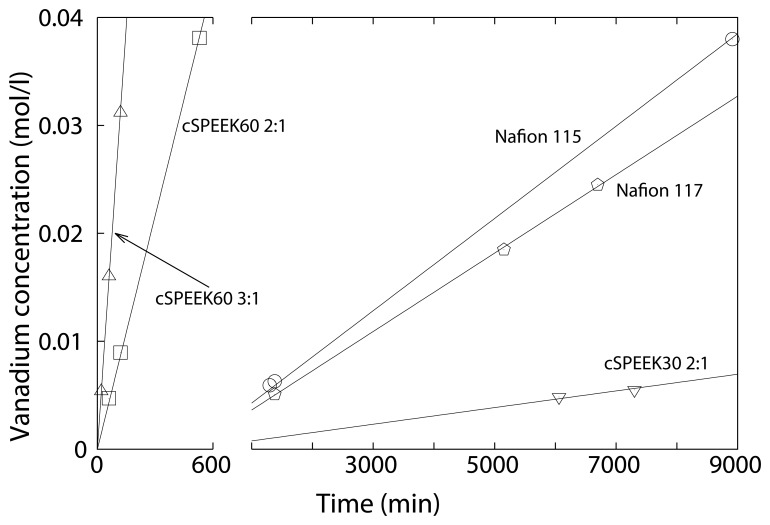
VO^2+^ crossover through cSPEEK membranes cured at 200 °C and Nafion membranes sample code: cSPEEK60 2:1 = crosslinked SPEEK with a crosslinking time of 60 min and a SPEEK:crosslinking ratio of 2:1).

The vanadium permeability of the synthesized and commercial membranes were studied. This was done in a diffusion cell, which consists of two half-cells separated by the membrane under investigation. Figure 13 depicts the increase of vanadium concentration in the MgSO_4_ compartments over time, where Table 2 depicts the permeability of VO^2+^ through these membranes.

**Table 2:** Ion exchange capacity (IEC; meq/g) and vanadium permeability (cm^2^ s^−1^) of observed membranes (sample code: cSPEEK60 2:1 = crosslinked SPEEK with a crosslinking time of 60 min and a SPEEK:crosslinking ratio of 2:1).

**Membranes**	**IEC**			**Permeability**	
	
	**Non-crosslinked**	**150 °C**	**200 °C**	**Non-crosslinked**	**200 °C**
cSPEEK60 3:1		1.60	2.05		6.7 × 10^−6^
cSPEEK60 2:1		1.43	1.97		4.8 × 10^−6^
cSPEEK30 2:1		1.12	1.34		5.7 × 10^−8^
Nafion 117	0.97 [36]			1.9 × 10^−6^	
SPEEK	2.24			∼	

It can be seen that the vanadium permeability decreased with decreasing the amount of SPEEK (*i.e.*, increasing the crosslinking). However, the vanadium permeability remains still higher than through a Nafion 117 membrane. This may be a result of the highly sulfonated SPEEK, which has a high water uptake. To overcome this issue, the ratios of crosslinked ${\text{SO}}_{3}^{-}$ groups to the non-crosslinked ${\text{SO}}_{3}^{-}$ groups had to be adjusted. This was done by decreasing the crosslinking time at 200 °C from 60 to 30 min. By this, the synthesized membrane (cSPEEK30 2:1) exhibited a vanadium permeability 100 times lower than Nafion 117 (Table 2). However, the FT-IR spectroscopy of the cSPEEK30 2:1 has not shown a significant increase of the sulfonate ester bonds. This might be due to the low resolution an FT-IR can offer on such intermediate crosslinked material. Due to that, other analysis methods, like Solid-State-NMR, could be of great help to understand the extent of crosslinking.

The reason for the low vanadium cross over in the case of the crosslinked SPEEK lies in its glassy properties and rigid backbone. Furthermore, the dead end hydrophilic channels of SPEEK also decrease the vanadium permeability, since these are not as well interconnected as in the case of Nafion.

## 4. Conclusions

Highly sulfonated crosslinked SPEEK membranes for a Vanadium/Air-RFB application were prepared as described elsewhere [22,23]. For this reaction, two crosslinking pathways were proposed and proven by FT-IR, IEC and proton conductivity measurements. These are the crosslinking of the 1,4-benzenedimethanol on ${\text{SO}}_{3}^{-}$ groups at temperatures of ≤150 °C and the crosslinking of the 1,4-benzenedimethanol on the backbone of the SPEEK unit at a temperature of 200 °C. By applying a crosslinking temperature of 200 °C, the proton conducting groups ( ${\text{SO}}_{3}^{-}$) can be preserved. Furthermore, we have investigated the water diffusion through the crosslinked SPEEK membranes by Pulsed Field Gradient (PFG) NMR measurements. Here, the anisotropy coefficient indicates an orientation of water channels, preferably perpendicular to the membrane plane. However, the water diffusion coefficient increases in the membranes with increasing temperature and time. This could be explained by the preservation of the sulfonic acid groups (due to backbone crosslinking) and the hydrophilicity of the channels. However, since the highly proton-conductive SPEEK shows a high vanadium permeability, the ratio of crosslinked and non-crosslinked ${\text{SO}}_{3}^{-}$ groups was balanced in order to decrease the vanadium crossover. This adjustment was done by varying the crosslinking time. The final membrane exhibits a proton conductivity of 27.9 mS/cm and a 100 times lower vanadium permeability than Nafion 117.
